# Spectrum of variations in *dog-1/FANCJ* and *mdf-1/MAD1* defective *Caenorhabditis elegans* strains after long-term propagation

**DOI:** 10.1186/s12864-015-1402-y

**Published:** 2015-03-18

**Authors:** Maja Tarailo-Graovac, Tammy Wong, Zhaozhao Qin, Stephane Flibotte, Jon Taylor, Donald G Moerman, Ann M Rose, Nansheng Chen

**Affiliations:** Department of Molecular Biology and Biochemistry, Simon Fraser University, V5A 1S6 Burnaby, BC Canada; Department of Zoology, University of British Columbia, V6T 1Z4 Vancouver, BC Canada; Department of Medical Genetics, University of British Columbia, V6T 1Z3 Vancouver, BC Canada; Current affiliation: Centre for Molecular Medicine and Therapeutics; Child and Family Research Institute, Vancouver, BC Canada; Current affiliation: Treatable Intellectual Disability Endeavour in British Columbia, Vancouver, BC Canada

**Keywords:** C. elegans, Whole genome sequencing (WGS), oligonucleotide array Comparative Genomic Hybridization (oaCGH), Mutation accumulation (MA), Genomic variation (GV), Spindle assembly checkpoint (SAC), dog-1/FANCJ, G-quadruplex (G_4_) structure

## Abstract

**Background:**

Whole and partial chromosome losses or gains and structural chromosome changes are hallmarks of human tumors. Guanine-rich DNA, which has a potential to form a G-quadruplex (G_4_) structure, is particularly vulnerable to changes. In *Caenorhabditis elegans*, faithful transmission of G-rich DNA is ensured by the DOG-1/FANCJ deadbox helicase.

**Results:**

To identify a spectrum of mutations, after long-term propagation, we combined whole genome sequencing (WGS) and oligonucleotide array Comparative Genomic Hybridization (oaCGH) analysis of a *C. elegans* strain that was propagated, in the absence of DOG-1 and MDF-1/MAD1, for a total of 470 generations, with samples taken for long term storage (by freezing) in generations 170 and 270. We compared the genomes of F_170_ and F_470_ strains and identified 94 substitutions, 17 InDels, 3 duplications, and 139 deletions larger than 20 bp. These homozygous variants were predicted to impact 101 protein-coding genes. Phenotypic analysis of this strain revealed remarkable fitness recovery indicating that mutations, which have accumulated in the strain, are not only tolerated but also cooperate to achieve long-term population survival in the absence of DOG-1 and MDF-1. Furthermore, deletions larger than 20 bp were the only variants that frequently occurred in G-rich DNA. We showed that 126 of the possible 954 predicted monoG/C tracts, larger than 14 bp, were deleted in *unc-46 mdf-1 such-4; dog-1* F_470_ (JNC170).

**Conclusions:**

Here, we identified variants that accumulated in *C. elegans’* genome after long-term propagation in the absence of DOG-1 and MDF-1. We showed that DNA sequences, with G_4_-forming potential, are vulnerable to deletion-formation in this genetic background.

**Electronic supplementary material:**

The online version of this article (doi:10.1186/s12864-015-1402-y) contains supplementary material, which is available to authorized users.

## Background

Genome integrity is crucial for survival of all living organisms. Chromosomal instability (CIN), marked by whole or segmental aneuploidy is hallmark of human tumors, and can drive abnormal proliferation of cancer cells [1]. In *Caenorhabditis elegans*, *mdf-1(gk2)* has an essential *mdf-1/MAD-1* component of the spindle assembly checkpoint (SAC) missing and this leads to accumulation of genetic errors and ultimately death by the third generation [2]. The checkpoint prevents CIN by inhibiting anaphase-promoting complex/cyclosome (APC/C), and delaying anaphase onset until all the chromosomes have achieved proper attachment to the spindle [3].

While MDF-1 prevents both loss and gain of whole chromosomes during mitosis, DOG-1 prevents segmental aneuploidies by ensuring proper replication of guanine(G)-rich DNA [4-6]. G-rich DNA can adopt a four-stranded helical G-quadruplex (G_4_) DNA structure [7-9], which can pose a barrier to replication fork progression if left unresolved. The ability to form G_4_ structures makes the corresponding G-rich DNA sequences particularly vulnerable to chromosomal rearrangements. Studies using *C. elegans*, as a model organism, were the first to show the striking genomic instability of G-rich DNA sequences when DOG-1, a functional ortholog of the deadbox helicase FANCJ [10], was non-functional [4]. When DOG-1 is functional, G-rich DNA sequences are stable and deletions affecting these regions are not observed [4,5]. Genome-wide bioinformatic analysis of the human genome had identified more than 300,000 DNA sites with G_4_-forming potential [11,12]. In humans, mutations in *FANCJ/dog-1* have been identified in Fanconi anemia (FA) complementation group J patients [13-15], which is a severe, autosomal recessive, disorder with increased spontaneous and DNA crosslink-induced CIN showing a wide range of clinical manifestations [13], and also in early onset breast cancer patients [16,17].

Knowledge of the mutational spectra is crucial for deciphering the cause of heritable genetic disorders as well as the progression of events relevant to cancers. Traditionally, analyses of mutation spectra and rates have been based on a small portion of phenotypically and molecularly detectable loci. In *C. elegans*, the mutational spectrum of *dog-1(gk10)* (knockout allele of *dog-1*) strains, was analyzed using either PCR-based assays [4,10,18] or oligonucleotide array Comparative Genomic Hybridization (oaCGH) [5,6,19]. The rapid advances in “next-generation” DNA sequencing technologies now allows us to perform comprehensive genome-wide analyses of mutational spectra by sequencing whole genomes [20-23].

Here we undertook a whole genome approach in order to analyze mutational events in a *C. elegans* strain that is defective for both *mdf-1/MAD1* and *dog-1/FANCJ*. This strain was propagated for 470 generations and samples were stored frozen at generations 170 and 270. Phenotypic analysis of the strain revealed striking fitness recovery, indicating that accumulated mutations cooperate to bypass the MDF-1 checkpoint requirement and thus achieve long-term population survival. We performed whole-genome sequencing (WGS) and oaCGH analyses of the strain at three different generations (170, 270 and 470). We identified substitutions, InDels, and copy number variants (CNVs) larger than 20 bp, and compared their accumulation over the generations. We showed that only deletions, which are larger than 20 bp, frequently initiated in G-rich DNA (88% of all of the deletions). Consistent with the fitness recovery observed in this strain, rather than a decline in fitness, the mutation spectrum reported here reflects variants that are either advantageous or neutral in this specific genetic background.

## Results and discussion

### Whole genome analysis

**Figure 1:**
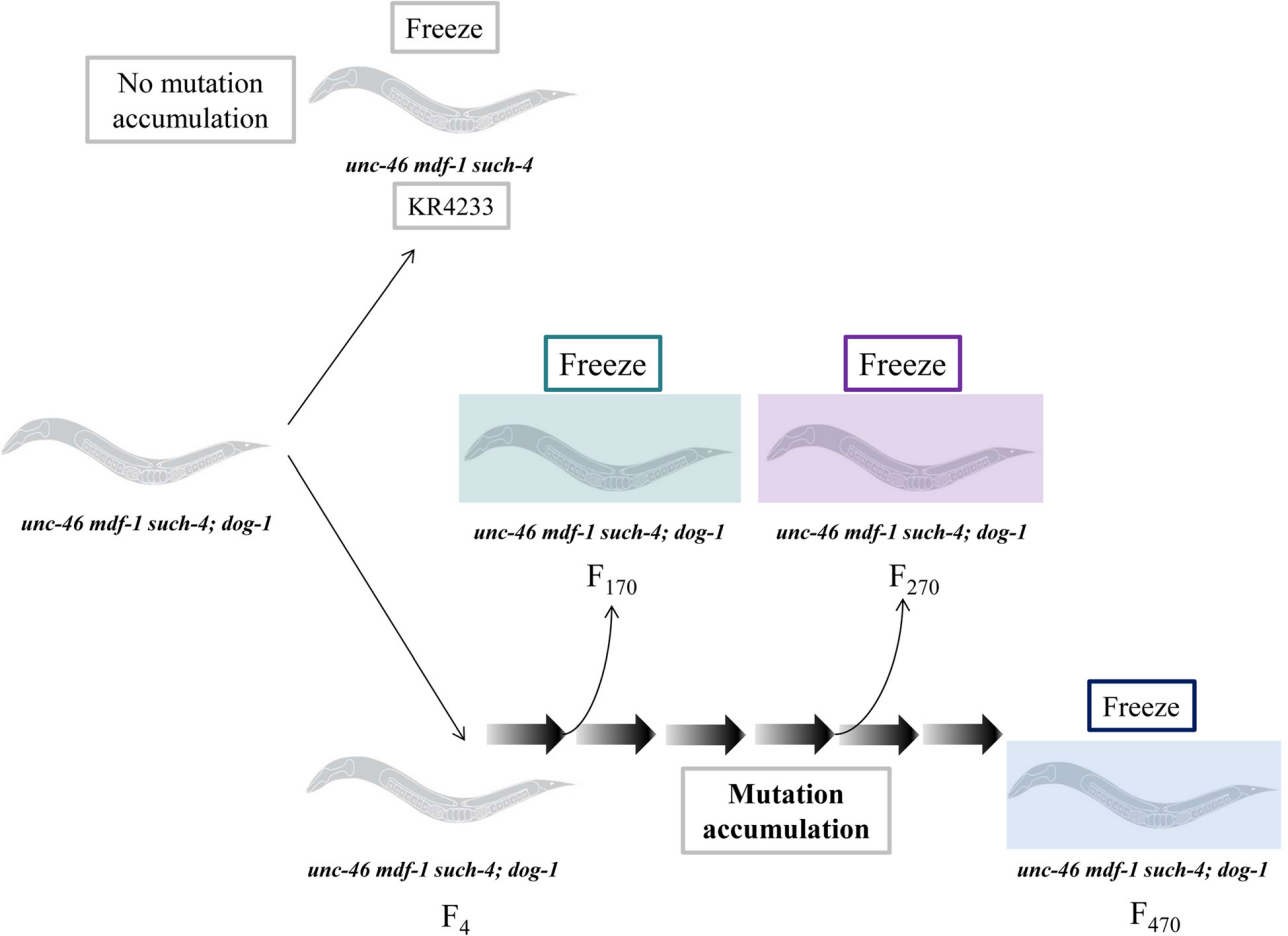
**A schematic representation of the long-term propagation of** ***unc-46 mdf-1 such-4; dog-1*** **homozygotes for 470 generations.** We isolated a suppressor (*such-4*) in F_4_ from the only plate that we set up that propagated in the absence of MDF-1 and DOG-1 for longer than three generations. We generated a strain (KR4233) by crossing away *dog-1(gk10)*. A second line was propagated for the total of 470 generations. The worms were frozen at generations 170 (green), 270 (purple) and 470 (blue). Note that *unc-46* (used as a visible marker) is tightly linked to *mdf-1(gk2)* in all of our strains.

In order to propagate *mdf-1; dog-1* homozygous worms, it is first necessary to isolate a suppressor of *mdf-1(gk2)* sterility and lethality that occurs in the double mutant [24]. Previously, *such-4(h2168)* was isolated, which allowed propagation of *mdf-1; dog-1* beyond the third generation (Figure 1) [24]. The *such-4* suppressor allows for an approximately five-fold increase in fertile hermaphrodite progeny of *mdf-1; dog-1* [24]. This increase in fertility occurs in the generations immediately after isolation of the suppressor. We outcrossed one worm to obtain KR4233 [*unc-46(e177) mdf-1(gk2) such-4(h2168)*] [24,25] (Figure 1). A second line was isolated and maintained in the *dog-1(gk10)* background for 470 generations, with storage by freezing at generations 170 and 270 (Figure 1). To mark the presence of *mdf-1(gk2)*, *unc-46(e177)* (a visible marker) was used, which is present in all of our strains (Figure 1). While propagating *unc-46 mdf-1 such-4; dog-1* homozygotes, we observed a further increase in reproductive fitness. This increase was significant, 59% of F_470_*unc-46 mdf-1 such-4; dog-1* progeny develop into fertile hermaphrodites, compared to only 10% of the *unc-46 mdf-1 such-4* progeny and 2% of *unc-46 mdf-1* mutants. Detailed phenotypic analysis of these strains as well as genetic dissection of suppressors has recently been reported [26]. To identify the genomic variations (GVs) that had accumulated in *mdf-1 such-4; dog-1 F*_*470*_ worms after long-term propagation, the genome was sequenced to a depth of 70× genome equivalents and aligned to the *C. elegans* reference genome WS235 available at WormBase [27]. We also sequenced the *unc-46 mdf-1 such-4; dog-1* strains that were frozen at generations 170 and 270 and compared the progress of mutation accumulation (Additional file 1: Figure S1).

### Single Base Substitutions (SBSs) do not occur within G_4_-DNA

**Figure 2:**
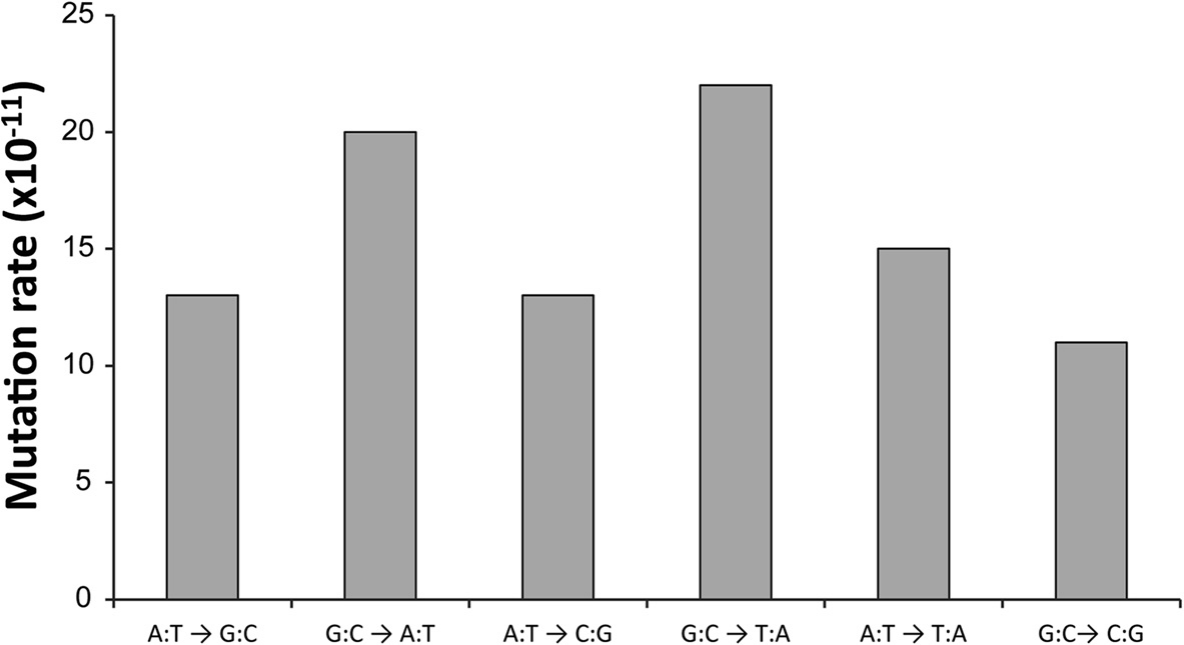
**Mutation rate estimates.** The variants analyzed are the 94 SBSs that were identified between generations 170 and 470.

We used the variant caller VarScan2; version 2.3.2 [28] to identify 525 homozygous SBSs that occurred with a variant frequency (VF) of 90% or higher in the *mdf-1 such-4; dog-1*_F470_ genome (Additional file 2: Table S1A). All 42 tested substitutions were confirmed by Sanger re-sequencing (30 were randomly selected; 12 additional substitutions were confirmed by re-sequencing as a result of being adjacent to randomly selected SBSs or as being non-randomly selected as candidates in later analyses), indicating a false positive rate of less than 5%. WGS analysis of the *unc-46 mdf-1 such-4; dog-1* strain, which had been frozen at generation 170 (Figure 1), revealed that the majority (431/525) of the SBSs present in generation 470 are also present in generation 170 (Additional file 1: Figure S1A; Table 1). However, we did observe that 32 additional substitutions had accumulated between F_170_ and F_270_, and 62 more between F_270_ and F_470_ (Table 1). Large number of substitutions observed in F_170_ indicate the possibility that the original *unc-46 mdf-1 such-4; dog-1* strain (Figure 1) had a large number of single nucleotide differences from the reference genome (WS235). One way to test this possibility is to estimate the mutation rates based on available data. If we consider the 94 substitutions that had accumulated in 300 generations, between F_170_ and F_470_, we estimate the rate of μ_bs_ = 3.1 × 10^-9^/base/generation. This estimate is similar to the previously reported spontaneous rate of base substitution in *C. elegans*, 2.7 ± 0.4 × 10^−9^ /base/generation [21] and other model organisms, 3.5 × 10^−9^ /base/generation in *Drosophila melanogaster* [23] and 7.1 ± 0.7 × 10^−9^ /base/generation in *Arabidopsis thaliana* [22]. Furthermore, analysis of the 94 substitutions on mutation bias revealed very similar mutation spectrum to the spontaneous mutation spectra in N2 (*C. elegans* wild-type) [21] (Figure 2), and our analysis of transition bias (transition/transversion – Ts/Tv – base substitution ratios) revealed the Ts/Tv ratio = 0.54, which is within the range observed for spontaneous mutations in multiple mutation accumulation lines (average 0.45; range 0.19 – 0.79) [21]. Therefore, the similarity of the substitution rate over the last 300 generations to the previously reported μ_bs_ in N2 strongly implies that the majority of the 431 substitutions identified in the F_170_ generation are variants originally present in the starting strain; therefore, we focused our analysis on GVs that had occurred between F_170_ and F_470_ (Additional file 2: Table S1B).

**Table 1:** **Summary of the variants identified in the** ***unc-46 mdf-1 such-4; dog-1*** **strains at generations F**_170_ **, F**_270_ **and F**_470_

	**SBSs**	**InDels ≤ 20 bp**	**Duplications**	**Deletions >20 bp**
F170	431	133	3	57
F270	32	8	3*	45
F470	62	9	– 1	94
Total F170-F470	94	17	3	139

*Two new duplications and amplification of the *cyb-3* locus to three copies.

Previous studies showed that G_4_ DNA secondary structure is mutagenic in the absence of DOG-1 [4-6,19]. Using the G_4_ DNA signature (G_3+_N_1-7_G_3+_N_1-7_G_3+_N_1-7_G_3+_), we identified 2,372 such sites in *C. elegans*’ genome (Additional file 2: Table S2). Next, we tested to see if any of the 94 substitutions occurred within the G_4_ DNA signature sequence and observed that none of them did (Additional file 2: Table S1B), indicating that the identified substitutions most likely arose spontaneously and were not due to lack of functional DOG-1.

### Small InDels do not occur within G_4_-DNA

We applied VarScan2 [28] to identify 150 homozygous InDels that were 20 bp or less in *mdf-1 such-4; dog-1*_F470_ (Additional file 2: Table S3A). We randomly selected 25 of the InDels and confirmed all 25 by Sanger re-sequencing, indicating a false positive rate of less than 5% (Additional file 2: Table S3A). Analysis of InDels in the *unc-46 mdf-1 such-4; dog-1* strains propagated for 170 and 270 generations (Additional file 1: Figure S1B) revealed that 88% of the F_470_ InDels (133/150) existed in the F_170_ generation (Table 1), which again indicates that the majority of InDels were already present in our starting strain. In fact, we only observe eight additional InDels in F_270_ (Table 1) and another nine InDels accumulated between F_270_ and F_470_ (Table 1). Based on the last 300 generations of propagation, we estimate a mutation rate for InDels to be 17/(1 × 10^8^_bases_ × 300_generations_) = 5.7 × 10^-10^/base/generation. The ratio of InDels to SBSs observed was 0.18 or one InDel per 5.5 substitutions, which is much lower than the 1.31 ratio reported previously in *C. elegans* [29]. However, the lower number of InDels to SBSs that we observe in the *unc-46 mdf-1 such-4; dog-1* background is comparable to analyses in yeast [30], *A. thaliana* [22] and human [31]. Namely, WGS analysis in yeast had revealed ratio of InDels to SBSs of ∼ 0.03 [30], which was consistent with previous findings of one InDel per 33 SBSs [20]. Furthermore, analysis on spontaneous occurrence of InDels in *A. thaliana* revealed the ratio of 0.13 of InDels to SBSs [22]. It may be possible that the small number of InDels, over the last 300 generations, in our strain may be a result of a mutation that was acquired by propagation; however, it may also be that the spontaneous mutation rate of InDels in *C. elegans* is comparable to that of other organisms.

Next, we tested to see if any of the 17 InDels occurred within the G_4_ DNA signature sequence and observed that none of them did (Additional file 2: Table S3B). Thus, we believe that these InDels arose spontaneously and are not due to a lack of functional DOG-1.

### Duplications do not occur within G_4_-DNA

Analysis of gene copy-number variant accumulation after long-term propagation in *C. elegans* using the oaCGH has provided evidence for a high rate of spontaneous gene duplications in this multi-cellular eukaryote [32]. Previously, using the oaCGH we showed that the *such-4* suppressor genome contains a large tandem duplication on Chromosome V (LGV) [6]. Here, we used both Pindel [33] and oaCGH [34], and identified four sites with copy number increases in the *mdf-1 such-4; dog-1*_F470_ genome (Additional file 2: Table S4A), including a previously identified large tandem duplication [6]. One of the duplications involves a two-copy addition, making the final count of five duplication events and four different duplication sites. Analysis of the CNVs in the F_170_ and F_270_ genomes captured a dynamic property of duplications (Additional file 1: Figure S1C). In F_170_, we observed three duplications (Additional file 2: Table S4A). One is the large tandem duplication located on LGV that amplifies 62 protein-coding genes, which we have described previously [6]. In generation F_270_, we detected duplications of two new loci, as well as further amplification of the LGV region to three copies (triplication) (Additional file 1: Figure S1C and Additional file 2: Table S4A). In F_470_, we did not find any new duplications (Additional file 2: Table S4A), but did observe that the duplication on LGI was lost, resulting in a wild-type copy number for this region (Additional file 2: Table S4A). Thus, the LGV duplication exemplifies the property of duplications to further amplify; while LGI duplication shows that a duplicated region can revert back to a normal copy number.

The gene duplication rate for *C. elegans* was recently estimated to be 3.4 × 10^-7^/**gene**/generation [32]. Our data, based on the last 300 generations (from F_170_ and F_470_), also revealed a comparably high rate of gene duplication (6.5 × 10^-7^/**gene**/generation), specific to the *unc-46 mdf-1 such-4; dog-1* background, when a large duplication on LGV is excluded from the analysis. The LGV duplicated region contains 62 protein-coding genes, including *cyb-3* [6]. We observed a correlation between increased dosage of CYB-3 and a striking fitness increase in our strains; thus, our experimental protocol selected for LGV amplification (detailed experimental analysis on these findings has been recently published [26]). Importantly, like SBSs and InDels, none of the duplications occurred in the vicinity of the G_4_ DNA signature sequences (Additional file 2: Table S4B), also indicating that these CNVs arose spontaneously and were not due to a lack of functional DOG-1.

### Deletions frequently initiate at G_4_ DNA sites

The major type of mutation observed, in the absence of DOG-1, is a deletion of 300 bp or smaller, initiating at either the 5′-end of C- or the 3′-end of G-tracts [4,5,10,19]. Using the unique alignments generated by Novoalign and Pindel [33], we identified 183 homozygous deletions larger than 20 bp in the *mdf-1 such-4; dog-1*_*F470*_ genome (Additional file 2: Table S5A), including the known *mdf-1(gk2)* and *dog-1(gk10)* deletions. We randomly selected 28 of the deletions, and confirmed all of them using PCR (Additional file 2: Table S5A). To identify deletions, which may have been missed, we also used oaCGH [34]. We confirmed all of the deletions, predicted by Pindel, that were covered by oaCGH probes and identified an additional 13 deletions not detected by Pindel (Additional file 2: Table S5A), making the final count 196 homozygous deletions in the *mdf-1 such-4; dog-1*_*F470*_ genome (Additional file 2: Table S5A).

We observed 57 deletions in F_170_, indicating that 139 deletions had accumulated in 300 generations (between F_170_ and F_470_) propagated in the absence of MDF-1 and DOG-1 (Additional file 1: Figure S1D and Table 1). We found that the majority of the deletions (123 of 139) initiated in G_4_ DNA (Additional file 2: Table S5A). Previous analysis in *C. elegans* [32] estimated the spontaneous rate of deletions to be 2.2 × 10^-7^/**gene**/generation respectively. The 139 deletions accumulating between the generations F_170_ and F_470_ affect 19 protein-coding genes which allowed us to calculate the mutation rate of deletions over the 300 generations to be 19/(20,400_protein-coding genes_ × 300_generations_) = 3.1 × 10^-6^/**gene**/generation in the *unc-46 mdf-1 such-4; dog-1* strain, which is approximately 10-fold higher than the estimated rate in N2 [32]. This is similar to the estimated forward mutation frequency of *eT1*-balanced lethal mutations in *dog-1(gk10)* background [6]. To determine if the elevated mutation rate of deletions is due to DOG-1 deficiency, we compared mutation rates in non-G_4_ sites versus mutation rate in the G_4_ sites. While the mutation rate of deletions affecting the non-G_4_ sites, 4.9 × 10^-7^/**gene**/generation or 5.0 × 10^-10^/base/generation, is comparable to the previously reported spontaneous rate of deletions [32]; the mutation rate based on G_4_ sites, 1.7 × 10^-4^ /**G**_**4**_**site**/generation, illustrates the striking vulnerability of these DNA regions when DOG-1 is absent. Therefore, deletions larger than 20 bp are the only variants in the *unc-46 mdf-1 such-4; dog-1* strain that frequently occurred in the G_4_-DNA sites and had significantly higher mutation rate than the spontaneous rate reported previously for the strains with normal DOG-1 function.

### monoG/C tracts larger than 14 bp are frequently deleted when DOG-1 is not functional

**Figure 3:**
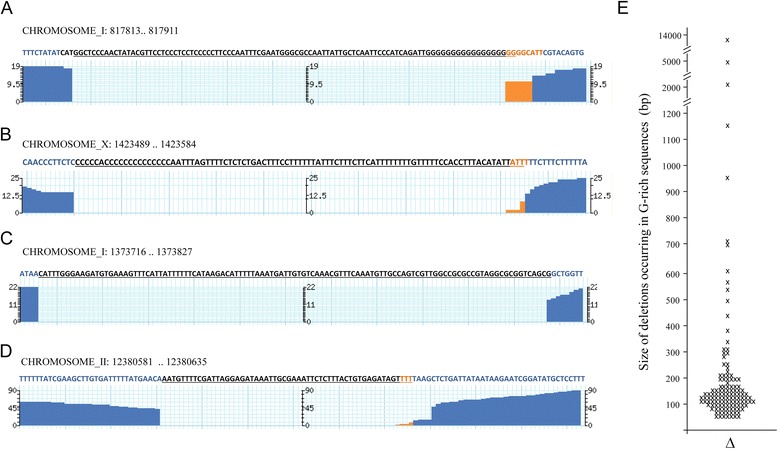
**The majority of large deletions initiates at G-rich DNA.** Figures **(A)** through **(D)** are Genome Browser snapshots of single deletions; x-axis represents genome location, while y-axis represents number of reads that cover the region; blue reads depict coverage of more than 10, while orange depicts 10 or less; the gaps depict no coverage, which is indicative of deletions. The reference sequence is depicted with deleted bases underlined. **(A)** A 99 bp deletion that initiates at G_20_ homopolymer. **(B)** A 96 bp deletion that initiates at G_14_TG_6_AGAAG_3_ sequence. **(C)** A 112 bp deletion that initiates at a G-rich sequence, G_2_N_3_G_2_N_5_G_2_N_2_G_2_. **(D)** A 55 bp deletion that initiates at a non G-rich sequence. **(E)** Size distribution of homozygous deletions that occur at G-rich sequences (n = 123).

In recent years, the G_4_ DNA has been implicated in diverse biological processes, such as gene expression [35] and DNA replication initiation [35]. Consistent with the established role of DOG-1, we found that the majority (114) of the homozygous deletions that we identified between F_170_ and F_470_ (139) initiated in the previously proposed G_4_ DNA signature G_3+_N_1-7_G_3+_N_1-7_G_3+_N_1-7_G_3+_ [5] (Additional file 2: Table S5B) where the G-tract was almost completely removed together with 5′ flanking DNA sequence (Figure 3A and B). In agreement with a previous study [5], we found that the majority of deletions initiate at monoG tracts larger than 14 bp (93 deletions) (Figure 3A), while 11 deletions initiate at monoG-like structures with no more than two nucleotides that interrupt the homopolymer (Figure 3B), and 10 deletions initiate at sequences that interrupt the homopolymers by three or more nucleotides (e.g., GGGtGGGGaagttatGGGaGGG) (Additional file 2: Table S5B). MonoG/C tracts larger than 14 bp have the highest potential of forming the G-quartet structure. In fact, we find here that the *unc-46 mdf-1 such-4; dog-1 F*_*470*_ genome has 13.2% of all the predicted monoG/C tracts larger than 14 bp deleted. An interesting question to be addressed with future research would be to determine how mutation rate changes with decreasing numbers of available targets. Furthermore, it would be also important to see how many of the G_4_-DNA sites could be deleted in a strain and still maintain viability of the animals.

To investigate whether there may be additional sequences, which are vulnerable in the absence of DOG-1, we analyzed the 25 deletions that do not initiate in G_4_ DNA signature sequences to see if there are common patterns. We found that eight of these deletions initiate at G-rich sequences that correspond to the G_2+_N_1-7_G_2+_N_1-7_G_2+_N_1-7_G_2+_ signature (four stretches of two or more guanines, alternated with one to seven nucleotides of any type), while one had a G_2+_N_1-7_G_2+_N_1-7_G_2+_ signature at the breakpoint (Figure 3C and Table 2). Although, it may be possible that our strain had gained a mutation in an unknown gene important for genome stability, it is also possible that additional DNA sequences may be vulnerable to rearrangements in the absence of DOG-1.

**Table 2:** **Schematic representation of the nine deletions that initiate at G-rich regions within sequences that deviate from the G4 DNA signature, G**_3+_ **N**_1-7_ **G**_3+_ **N**_1-7_ **G**_3+_ **N**_1-7_ **G**_3+_

**LG**	**Locus**	**Signature**
I	464950..465071	G_2_NG_12_
I	815471..815611	G_3_NG_3_NG_3_N_2_G_2_
I	3747905..3748027	G_2_NG_2_NG_2_NG_2_NG_2_NG_2_NG_2_NG_2_NG_2_
I	1373716..1373827	G_2_N_3_G_2_N_5_G_2_N_2_G_2_
II	12687460..12687546	G_2_NG_14_NG_2_
III	460436..460942	G_11_
IV	3298588..3298711	G_14_
V	17778167..17778449	G_3_NG_3_NG_3_N_5_G_2_NG_2_NG_3_
X	2802061..2802223*	G_2_NG_3_N_6_G_3_

*Analysis of the sequences revealed that nine match the G_2+_N_1-7_G_2+_N_1-7_G_2+_N_1-7_G_2+_ signature, while one (marked with *) matches G_2+_N_1-7_G_2+_N_1-7_G_2+_ signature at the breakpoint.

Previously, it was found that the deletion sizes in viable lines detected by PCR [4,18] or oaCGH methods [5,6] were predominately smaller than 300 bp. In this study, the 123 deletions that occurred at G-rich sequences ranged between 49 and 10,228 base-pairs with the majority of the deletions (86%) removing less than 300 bp (Figure 3E). These findings are in agreement with the deletion distribution sizes revealed by the study of a 69 G-tract deletion set [5]. However, larger deletions, initiating at G-tracts, have been recovered as lethal mutations [6], consistent with these regions containing essential genes [6]. We also found that 16 deletions, which occurred at non-G-rich sites, removed small regions of less than 300 bp in size (Additional file 2: Table S5B).

### 101 protein-coding genes are affected by the 253 GVs

To determine an effect of all the identified variants on protein-coding genes, after long-term propagation in the absence of MDF-1 and DOG-1, we used CooVar [36], a tool developed by our group for annotating variants. Many of the G-rich DNA sites are located in close proximity to protein-coding genes in *C. elegans* [19,37]*.* Using CooVar [36], we predicted that 19 genes would be affected by 18 of the deletions that had accumulated between generations F_170_ and F_470_ (Additional file 2: Table S6). The majority of the deletions (14) represent the first knockout alleles for those genes and thus provide a genetic resource for studying their functions (Additional file 2: Table S6). We found that the majority of the InDels are not located in protein-coding regions, and the one that affects a protein-coding gene is an in-frame deletion (Additional file 2: Table S6). We found that the majority of the detected SBSs are located within non-protein coding regions. However, there are 15 SBSs that fall within protein-coding regions that are predicted to result in missense mutations (Additional file 2: Table S6). In total, 66 protein-coding genes were affected by the duplications (Additional file 2: Table S6). The majority of the genes (62) are affected by the large tandem duplication on LGV (Additional file 2: Table S6). In addition to the 253 GVs that had accumulated between generations F_170_ and F_470_, we had also performed analysis of the effect on protein-coding genes of all the variants observed in the *mdf-1such-4; dog-1*_*F470*_ genome (Additional file 2: Table S7).

Mutations are a source of genetic variation that confers to an organism either advantage due to a beneficial change, disadvantage due to a deleterious alteration, or neither due to a neutral change. Considering the ~30-fold fitness recovery in this strain [26], the identified GVs are expected to be either advantageous or neutral in this genetic background. In fact we showed, in a parallel study, that three mutational events discovered in this strain cooperate to increase fitness when MDF-1 absent [26].

## Conclusion

In this study we undertook a genomics approach to identify variants that accumulate in the *C. elegans* genome after long-term propagation in the absence of two genome-guardians, DOG-1/FANCJ and MDF-1/MAD1. Combining WGS analysis with oaCGH analysis allowed us to comprehensively analyze both the small-scale variants (SBSs and InDels of 20 bp or smaller) and large-scale variants (CNVs larger than 20 bp). Freezing the strain, for long term storage, at three different time points, F_170_, F_270_ and F_470_, allowed us to compare and visualize mutation accumulation over the generations. We were able to estimate the mutation rates in this strain for different types of mutations over the 300 generations (F_170_ to F_470_). We observed a significantly elevated rate of deletions, larger than 20 bp, that initiated at G/C-rich DNA sequence. Our approach had allowed us to show that, in the absence of DOG-1, DNA sequences with G_4_-forming potential are vulnerable to deletion-formations. Previous studies were dependent only on PCR or oaCGH analysis; the two approaches are limited in their ability to detect small-scale variants like SBSs and InDels. Furthermore, genome analysis of the strains at F_170_, F_270_ and F_470_ allowed us to capture and visualize an intriguing property of CNVs (Additional file 1: Figure S1). We did not find any reversions of deletions, substitutions, or InDels. As expected, once fixed, these types of mutations are propagated indefinitely (Additional file 1: Figure S1). However, we captured a dynamic property of duplications: amplification of a region on LGV from one-to-two-to-three copies and reversion of the LGI duplication back to a normal copy number (Additional file 1: Figure S1C).

This is the first extensive analysis of a strain that had been propagated in the absence of DOG-1 helicase for hundreds of generations. We identified 954 monoG/C tracts larger than 14 bp in the *C. elegans* genome WS325; the polyG/C DNA sequence is the sequence with the highest potential of forming G-quartet. We showed that 13% of these 954 sites are deleted in the *mdf-1 such-4; dog-1*_*F470*_ genome. This finding raises an important question on the changes in mutation rate when number of mutagenic targets is decreased. Another important question is regarding to the role of G_4_ DNA in normal development. Recently, G_4_ DNA has been implicated in a variety of biological processes including telomere maintenance, gene expression, epigenetic regulation, and DNA replication [38]. One question to consider is how many G_4_ DNA sites could be removed from a genome yet still maintain viability of the animals.

## Methods

### *C. elegans* strains

The following mutant alleles were used in this work: *unc-46(e177), mdf-1(gk2), dog-1(gk10)*, and *such-4(h2168)*. The following strains were used in this work: KR4233 [*unc-46(e177) mdf-1(gk2) such-4(h2168)*] and KR3627 [*unc-46(e177) mdf-1(gk2) V/nT1[let-?(m435)])*]; VC13 [*dog-1(gk10)*]. Additional strains used in this work were generated in this study. Strains were maintained using standard protocol on nematode growth media (NGM) plates seeded with OP50 bacteria [39]. The strains were maintained at 20°C.

### Mutation accumulation procedure and phenotypic analysis

The first suppressor, *such-4*, was isolated as previously described [24]. Briefly, VC13 was backcrossed to N2 ten times to remove any mutations present in VC13. Then the outcrossed *dog-1(gk10)* males were used to construct *unc-46(e177) mdf-1(gk2) +/+ + nT1[let-X]; dog-1(gk10)/dog-1(gk10)*. Note that *unc-46(e177)* is linked *mdf-1(gk2)* and used as a visible marker to track *mdf-1(gk2)*. F_1_*unc-46 mdf-1* homozygotes (n = 40) were picked and plated individually and a single worm, from a plate containing fertile worms in the third generation, was isolated as a suppressor candidate (*such-4*) [24]. We outcrossed one worm from this strain to establish KR4233 *mdf-1(gk2) such-4(h2168)* [6,24], while we maintained a second line at 20°C for 470 generations (strain JNC170). Each generation 5 L_4_ hermaphrodites were transferred to a fresh plate. We also froze the worms at generations 170 (strain JNC168) and 270 (strain JNC169). The phenotypic analysis was performed as previously described [24].

### Whole genome sequencing and computational analysis of the *unc-46 mdf-1 such-4; dog-1*_*F470*_ genome

Genomic DNA was prepared from JNC170 following a standard protocol (http://genetics.wustl.edu/tslab/protocols/genomic-stuff/worm-genomic-dna-prep/) originally set up by the Andy Fire Lab. The library was prepared with average insert size of 300 bp and the genome was then sequenced using Illumina Solexa sequencing (at Canada’s Michael Smith Genome Sciences Centre) and 92,282,948 reads of 101 bp in length were obtained. The reads were then aligned to the *C. elegans* reference genome WS235 (hosted at WormBase) [27] in paired end manner (46,141,474 pairs) using the Novoalign alignment tool. 73,482,133 (79.63%) of the total reads were of base quality 30 or more and were uniquely mapped, generating 70-fold coverage of the genome.

SBSs were detected using the uniquely mapped reads and pileup2snp function of the variant caller VarScan2; version 2.3.2 [28]. We used SAMtools [40] to generate the mpileup file necessary as input for VarScan. We filtered out the SBSs that did not meet the following criteria: depth of coverage > 5 and ≤ 200, variant frequency ≥ 0.9 and base quality ≥ 30. After these filtering steps, we identified 776 substitutions in the *mdf-1 such-4; dog-1*_F470_ genome. However, comparison with the sequenced N2 strains from CGC and Horvitz lab (sequencing reads were kind gift from Dr. Bob Waterston) revealed 525 homozygous substitutions that are unique to *mdf-1 such-4; dog-1*_F470_ genome. The 251 SBSs were not included in our analysis as they did not accumulate during the course of our experiment. To determine rate of false positives in our set, we randomly selected 30 SBSs (Additional file 2: Table S1A), designed primers flanking the predicted substitution sites, amplified the fragments and re-sequenced using Sanger sequencing method at Genewiz, Inc. All 30 substitutions were confirmed by Sanger re-sequencing, suggesting a false positive rate of less than 5% (Additional file 2: Table S1A). Also, 12 additional substitutions were confirmed by re-sequencing as a result of being adjacent to randomly selected SBSs or as being non-randomly selected as candidates in later analyses.

InDels were detected using the uniquely mapped reads and pileup2indel function of the variant caller VarScan2; version 2.3.2 [28]. We used SAMtools [40] to generate the mpileup file necessary as input for VarScan2. We filtered out the InDels that did not meet the following criteria: depth of coverage > 5 and ≤ 200, variant frequency ≥ 0.9 and base quality ≥ 30. Additionally, we re-evaluated and filtered the output so that each co-ordinate corresponds to one variant. After these filtering steps, we identified 556 InDels in the *mdf-1 such-4; dog-1*_F470_ genome. r, comparison with the sequenced N2 strains from CGC and Horvitz lab (sequencing reads were kind gift from Dr. Bob Waterston) revealed 150 homozygous InDels that are unique to *mdf-1 such-4; dog-1*_F470_ genome. The 406 InDels were not included in our analysis as they did not accumulate during the course of our experiment. To determine rate of false positives in our set, we have randomly selected 25 InDels (Additional file 2: Table S3A), designed primers flanking the predicted InDel sites, amplified the fragments and re-sequenced using Sanger sequencing method at Genewiz, Inc. All of the 25 InDels were confirmed by Sanger re-sequencing, suggesting a false positive rate of less than 5% (Additional file 2: Table S3A).

These unique-mapping reads were then used, together with Pindel [33], to identify deletions. The final set of fixed/homozygous deletions was selected based on the following criteria: *a* = the number of unique reads supporting the breakpoints of the deletion; *b* = the number of reads within the deleted region; select the predicted deletion if $\frac{a}{a+b}$ is larger than 0.5 and the size of the deletion is larger than 20 bp. From the candidate deletions, 28 were randomly selected and the regions were PCR-amplified using the same genomic DNA that was used for the WGS and primers designed in the flanking regions of the computationally identified deletions. All of the 28 randomly selected predicted deletion sizes were confirmed using DNA electrophoresis gels, suggesting the rate of false positives of less than 5%.

### Whole genome sequencing and computational analysis of the *unc-46 mdf-1 such-4; dog-1*_*F1*70_ and *mdf-1 such-4; dog-1*_*F270*_ strains

The genomic DNA was prepared from JNC168 and JNC169 following a standard protocol (http://genetics.wustl.edu/tslab/protocols/genomic-stuff/worm-genomic-dna-prep/). The genomic DNA was sheared to generate 500 bp fragment and the library was prepared using the NEBNext® Ultra™ DNA Library Prep Kit for Illumina®. The library was then sequenced using Illumina Solexa sequencing (at Simon Fraser University) and 9,950,748 (F_170_) and 6,225,488 (F_270_) reads of 250 bp length were obtained. The reads were then aligned to the *C. elegans* reference genome WS235 (hosted at WormBase) [27] in paired end manner using the Novoalign alignment tool as described above to achieve 20-fold (F_170_) and 14-fold (F_270_) coverage. For both strains, we used Pindel [33] to identify deletions and duplications, and VarScan2; version 2.3.2 [28] to identify small InDels and substitutions, as described above. We also used sequenced N2 strains from CGC and Horvitz lab to remove variants existing in the N2 strain. Then, we compared the variants present in the F_170_ and F_270_ genomes with the ones identified in the F_470_ genome.

### oaCGH analysis of the *unc-46 mdf-1 such-4; dog-1* strains

To perform oaCGH analysis, we used the same genomic DNA from the *mdf-1 such-4; dog-1* lines (F_170_, F_270_ and F_470_) that were used for the WGS and the reference N2 DNA that was prepared following a standard protocol. oaCGH analysis was performed as described by Maydan and colleagues [41] with a newly designed microarray. The 3-plex microarray with design name 120618_Cele_WS230_JK_CGH was manufactured by Roche NimbleGen Inc. with each individual sub-array comprising 720 k 50-mer oligonucleotide probes. The filters used to select the probes primarily followed Maydan and colleagues [41] without focusing on coding regions in order to provide a more uniform coverage of the genome (WormBase release WS230). In regions where unique probes could not be designed selection filters were slightly relaxed in order to allow the inclusion of probes with possible cross-hybridization to at most one other location in the genome.

## Availability of supporting data

Whole Genome Sequencing (WGS) data, fastq files, for the three strains: JNC168, JNC169 and JNC170 are available in the NCBI Sequence Read Archive (SRA) (http://www.ncbi.nlm.nih.gov/sra) under the BioProject accession number SRP053517 (PRJNA275156) with the fastq files: (170_R1.fastq, 170_R2.fastq, 270_R1.fastq, 270_R2.fastq, 470_R1.fastq and 470_R2.fastq) under the SRR1797354 accession number.

## Additional files

Additional file 1: Figure S1. Mutation accumulation in *unc-46 mdf-1 such-4; dog-1* strains. Accumulation of mutations was visualized using Circos [42]. Note that due to the limited resolution, data points that occur close in the genome may appear as a single line or a single link. F_170_ chromosomes and variants originating at F_170_ are depicted in green; F_270_ chromosomes and variants originating at F_270_ are depicted in purple, while F_470_ chromosomes and variants unique to F_470_ are depicted in blue. The outer circle is a plot of all the variants present at a specific generation, while inner links are depicting propagation of the variants from one generation time-point to the next. The following variants are shown: (A) SBSs, (B) InDels, (C) Duplications, (D) Deletions. Additional file 2: Table S1A. Homozygous SBSs identified in the F_170_; F_270_ and F_470_ genomes. **Table S1B.** Homozygous SBSs identified after propagating *mdf-1; dog-1; such-4* for 300 generations. **Table S2.** Location of the G_3-5_ N_1-7_G_3-5_ N_1-7_G_3-5_ N_1-7_G_3-5_ sequences in the *C. elegans* genome. **Table S3A.** Homozygous InDels smaller than or equal to 20 bp identified in the F_170_; F_270_ and F_470_ genomes. **Table S3B.** Homozygous InDels smaller than or equal to 20 bp after propagating *mdf-1; dog-1; such-4* for 300 generations. **Table S4A.** Duplications identified in the F_170_; F_270_ and F_470_ genomes. **Table S4B.** Duplications identified after propagating *mdf-1; dog-1; such-4* for 300 generations. **Table S5A.** Homozygous deletions larger than 20 bp identified in the F_170_; F_270_ and F_470_ genomes. **Table S5B.** Homozygous deletions larger than 20 bp identified after propagating *mdf-1; dog-1; such-4* for 300 generations. **Table S6.** Accumulation of mutations predicted to disrupt protein-coding genes after 300 generations of propagation. **Table S7.** All mutations predicted to disrupt protein-coding genes in the *mdf-1; dog-1; such-4* F_470_ genome.

## Abbreviations

SAC: *S*pindle *a*ssembly *c*heckpoint
APC/C: *A*naphase *p*romoting *c*omplex or *c*yclosome
CIN: *C*hromosome *in*stability
WGS: *W*hole *g*enome sequencing
FA: Fanconi anemia
oaCGH: *o*ligonucleotide *a*rray *C*omparative *G*enomic *H*ybridization
G_4_: G-quartet
SBS: *S*ingle *b*ase *s*ubstitution
GV: Genome variant
