# Supplementary figures and images for: Spectrum of variations in dog-1/FANCJ and mdf-1/MAD1 defective Caenorhabditis elegans strains after long-term propagation
Source: BMC Genomics. 2015 Mar 18;16(1):210. doi: 10.1186/s12864-015-1402-y (PMC4369104; doi:10.1186/s12864-015-1402-y)

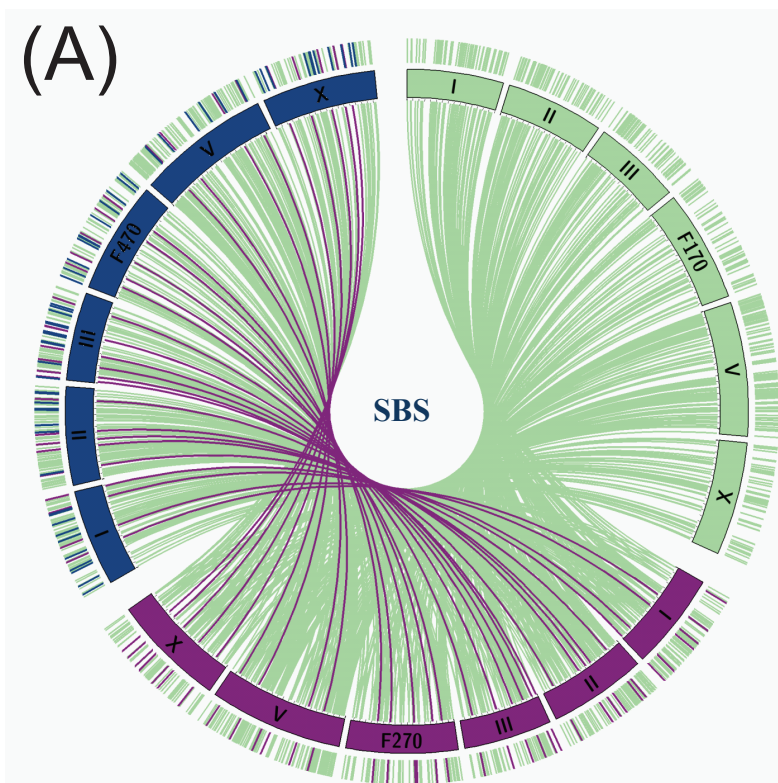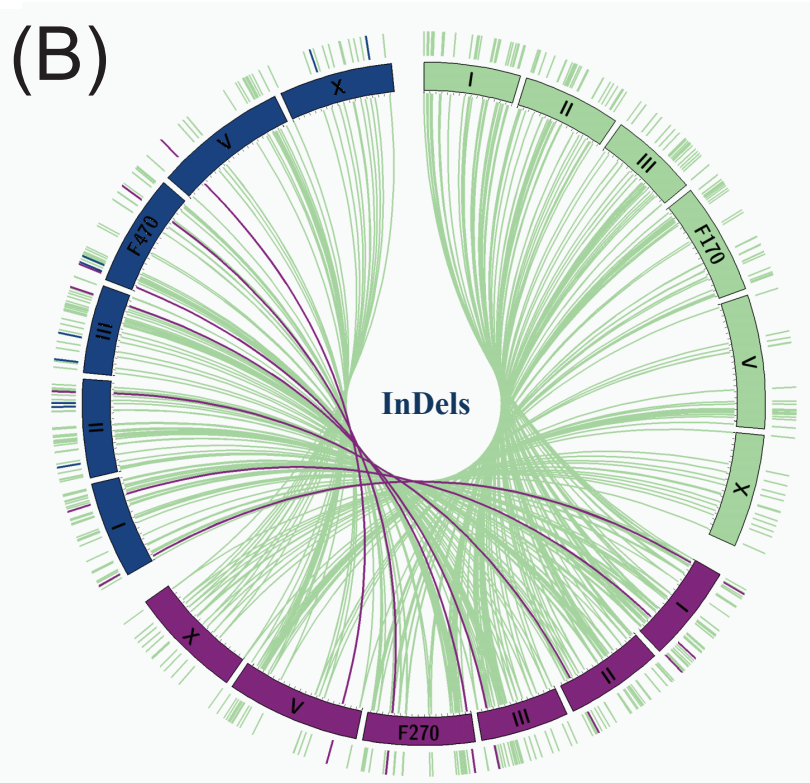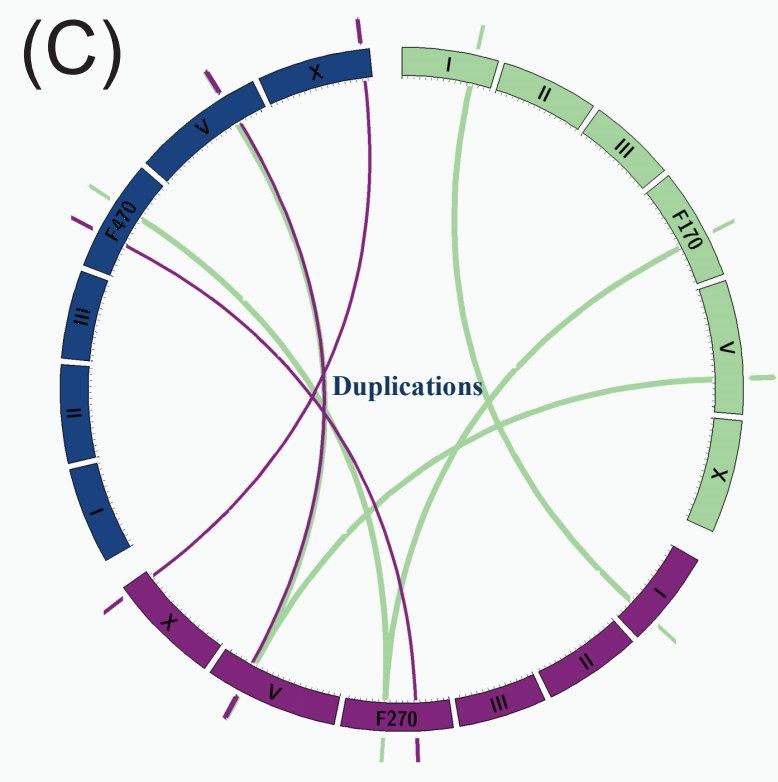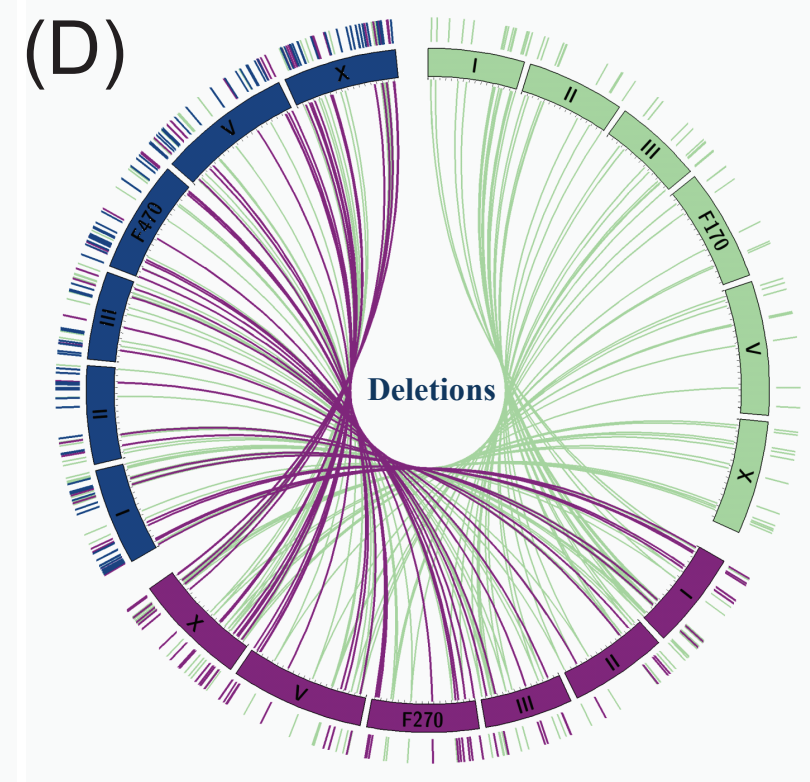

Supplement: Additional file 1: Figure S1. — Mutation accumulation in unc-46 mdf-1 such-4; dog-1 strains. Accumulation of mutations was visualized using Circos [42]. Note that due to the limited resolution, data points that occur close in the genome may appear as a single line or a single link. F170 chromosomes and variants originating at F170 are depicted in green; F270 chromosomes and variants originating at F270 are depicted in purple, while F470 chromosomes and variants unique to F470 are depicted in blue. The outer circle is a plot of all the variants present at a specific generation, while inner links are depicting propagation of the variants from one generation time-point to the next. The following variants are shown: (A) SBSs, (B) InDels, (C) Duplications, (D) Deletions. [file 12864_2015_1402_MOESM1_ESM.pdf]
